# Comparison of Long-Term Effect Between Direct and Indirect Bypass for Pediatric Ischemic-Type Moyamoya Disease: A Propensity Score-Matched Study

**DOI:** 10.3389/fneur.2019.00795

**Published:** 2019-07-31

**Authors:** Yahui Zhao, Junlin Lu, Shaochen Yu, Jiaxi Li, Xiaofeng Deng, Yan Zhang, Dong Zhang, Rong Wang, Hao Wang, Yuanli Zhao

**Affiliations:** ^1^Department of Neurosurgery, Beijing Tiantan Hospital, Capital Medical University, Beijing, China; ^2^Center of Stroke, Beijing Institute for Brain Disorders, Beijing, China; ^3^China National Clinical Research Center for Neurological Diseases, Beijing, China; ^4^Beijing Key Laboratory of Translational Medicine for Cerebrovascular Disease, Beijing, China

**Keywords:** moyamoya disease, direct bypass, indirect bypass, long-term outcome, pediatric MMD

## Abstract

**Objectives:** This study aimed to compare the postoperative risks and long-term effects between direct bypass surgery (DB) and indirect bypass (IB) surgery for pediatric patients with ischemic-type moyamoya disease (MMD).

**Method:** Pediatric patients (under or equal to 18 years old) who were diagnosed as MMD and given surgical treatments at our center between 2009 and 2015 were retrospectively reviewed from a prospective database. Pediatric hemorrhagic-type MMD patients and those who did not undergo digital subtraction angiography (DSA) were excluded. Patients who underwent DB were matched with patients who underwent IB using 1:1 propensity score matching. Postoperative complications, recurrent ischemic stroke events and modified Rankin Scale (mRS) scores at the last follow-up were compared between the matched pairs.

**Results:** A total of 223 pediatric patients were screened, and 138 patients (DB:34, IB:104) were considered for the propensity score match. Thirty four pairs were obtained. Nine patients had postoperative complications, including 6 (17.6%) in the DB group and 3 (8.8%) in the IB group (*P* = 0.476). The mean follow-up period was 71.9 ± 22.2 months for the DB group and 60.2 ± 24.3 months for the IB group (*P* = 0.041). Kaplan-Meier analysis showed a longer stroke-free time in the DB group than in the IB group (*P* = 0.025). At last follow-up, good neurological status (mRS ≤ 1) was achieved in 32 (94.1%) of the DB group and 34 (100.0%) of the IB group. MRS score at last follow-up were significantly lower than at time of admission (all pts: 1.09 ± 0.45 vs. 0.28 ± 0.51, *P* < 0.001; DB group: 1.12 ± 0.48 vs. 0.32 ± 0.59, *P* < 0.001; IB group: 1.06 ± 0.42 vs. 0.24 ± 0.43, *P* < 0.001).

**Conclusion:** Both techniques were effective in improving the neurological status of pediatric ischemic-type MMD patients, and direct bypass surgery might be more superior in preventing recurrent ischemic strokes in the short-term.

## Introduction

Moyamoya disease (MMD) is characterized by progressive stenosis and occlusion of the terminal internal carotid artery (ICA) and its branches, leading to severely compromised cerebral blood flow and subsequent ischemic or hemorrhagic strokes (1–3). A Japanese epidemiologic study reported a two-peak feature of onset age in MMD patients, affecting both children (5–9 years old) and middle-aged adults (around 40 years old) (4). Surgical revascularization has been recognized as the most effective treatment for MMD as it significantly reduces the risk of future strokes comparing to conservative management (2, 5, 6). Currently, the most adopted revascularization procedures include direct bypass (DB) and combined bypass surgery which involve anastomosis of superficial temporal artery (STA) and middle cerebral artery (MCA), and indirect bypass (IB) surgery which facilitates ingrowth of collaterals by attaching vascularized grafts to the cortical surface. The aforementioned surgeries have all been proved to be effective for MMD, and been applied in revascularization for both adult and pediatric MMD patients (5, 7–10).

Revascularization for pediatric MMD is not exactly the same as for adult MMD, as most of the pediatric patients manifest ischemic symptoms, while hemorrhagic events are commonly seen during disease process in adult patients (2, 11, 12), therefore, the main purpose of revascularization for pediatric MMD is to increase cerebral perfusion and prevent future brain ischemia. DB for children is technically more challenged, as donor and recipient vessels are often more delicate in pediatric patients than adults (11, 13, 14). On the other hand, IB, which is a much simpler technique, has been reported to provide abundant revascularization in pediatric MMD (15–17). Previous studies showed that in adult ischemic-type MMD patients, the direct bypass was more effective in preventing recurrent ischemic strokes than indirect bypass, despite the fact that similar long-term neurological outcomes were achieved by different surgical modalities (9, 18). However, the long-term effect of DB and IB in pediatric MMD had seldom been compared. Therefore, we conducted the current study to investigate the effect of DB and IB for ischemic-type pediatric MMD patients. To increase the comparability between patients who underwent different surgical modalities, we performed propensity score matching between patients who underwent DB and IB, then, surgical outcomes were compared between the matched groups.

## Methods

### Patients Selection

**Figure 1:**
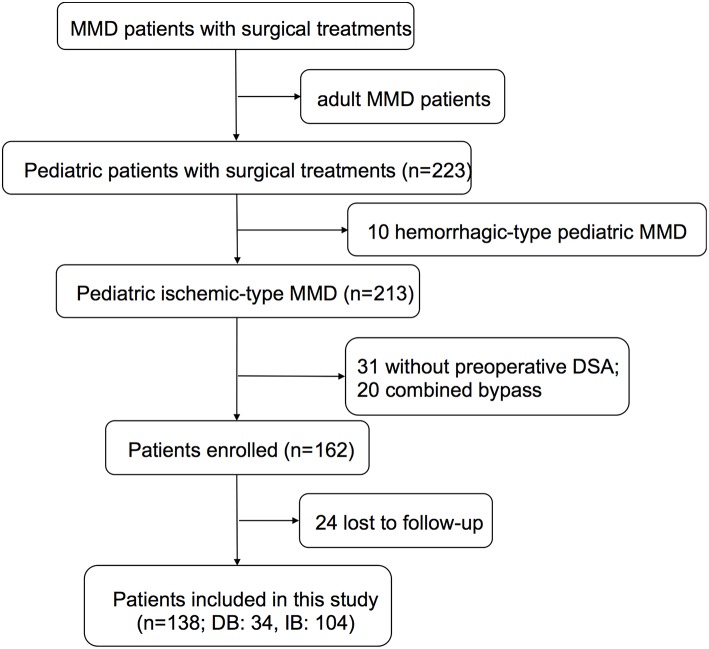
Flow diagram showing the selection of patients in the study.

Pediatric patients (under or equal to 18 years old) who were diagnosed as MMD and given surgical treatments at our center between 2009 and 2015 were retrospectively reviewed from a prospective database. The diagnosis of MMD was made according to the 2012 Guideline set by the Research Committee on the Pathology and Treatment of Spontaneous Occlusion of the Circle of Willis. Patients with moyamoya syndrome second to identified etiologies were ruled out. Adult MMD patients, pediatric hemorrhagic-type MMD patients, and patients who did not undergo digital subtraction angiography (DSA) were also excluded (Figure 1). This study was approved by the Institutional Review Board of Beijing Tiantan Hospital, Capital Medical University. Written consents were obtained at the time of admission for research use. For patients younger than 16 years old, parental consents were obtained; otherwise, written consents were obtained from patients themselves.

### Surgical Modalities

Two kinds of surgeries were included in this study, including DB and IB. DB was performed as end-to side anastomosis of STA branch to the cortical branch of MCA (M4 segment), by one single experienced surgeon at our center. IB included multiple bur hole surgery and encephaloduroarteriosynangiosis (EDAS), which were performed as attaching STA branch onto the cortical surface.

### Postoperative Managements and Complications

After surgery, all patients were given fluid therapy and blood pressure was monitored. Postoperative blood pressure (BP) was controlled with reference to preoperative basic BP and surgical modalities: for direct bypass surgery, postoperative BP was maintained slightly lower than or equal to basic BP; for indirect bypass surgery, it was maintained a bit higher than basic BP for fear of hypoperfusion. No antiplatelet agents were administered. Computed tomography (CT) and magnetic resonance imaging (MRI) were performed when patients had newly-developed symptoms during the postoperative period (within 7 days after the surgery in both DB and IB groups). Postoperative complications including intracerebral hemorrhage, intracerebral infarction, transient neurological deficits (which recovered fully before discharge with no sequela left and demonstrated no newly-developed lesion of infarction or hemorrhage on radiology), and seizures were recorded.

### Follow-Up Protocols

Patients were followed at the clinic or by telephone 3–6 months after surgery and annually thereafter by doctors who were blinded to the baseline information. Recurrent symptoms were recorded, including recurrent intracerebral hemorrhage, intracerebral infarction, transient ischemic attacks (TIAs), and seizures. Neurological status was evaluated by the modified Rankin scale (mRS).

### Statistical Analysis

Statistical analyses were performed using R statistical program (R studio; version 3.3.3). Propensity score matching was adopted in this study to overcome the imbalance and heterogeneity between different surgical modalities caused by the retrospective inclusion of patients. Based on logistic model, 1:1 propensity score matching using the “nearest” matching algorithm was performed with respect to age, sex, mRS at admission, Suzuki stage of the operated side, history of cerebral infarction, unilateral/bilateral involvement, and PCA involvement. Postoperative complications and long-term outcomes were then compared between the matched couples.

Categorical variables are presented as counts (with percentages) and continuous variables are presented as the means ± standard deviations. Grouped data were compared using *t*-test, chi-square test, Fisher's exam test, or Wilcoxon-Mann-Whitney rank-sum test as appropriate. Kaplan-Meier analysis was used to compare the stroke-free rate between different groups. A *P* < 0.05 was considered statistically significant.

Statistical power was calculated using G^*^power software (version 3.1). Sensitivity analysis showed that, for current sample size [given α error probability = 0.05, power (1-β error probability) = 0.8], the study was powered to detect a difference in proportions from 34.7 to 10.0% (estimated rate of postoperative complications and recurrent symptoms) between surgical groups. The effect size for the rank-sum test in this study was 0.62. *Post hoc* power analysis was used to calculate the statistical power (1-β) of comparison between matched pairs.

## Results

A total of 223 pediatric (≤18 years) MMD patients who underwent surgical revascularization at our center between 2009 and 2015 were retrospectively reviewed. Ten Patients with hemorrhagic-type MMD, 31 without preoperative DSA, 20 patients who underwent combined bypass (STA-MCA anastomosis and EDAS), and 24 patients who were lost to follow-up were excluded. Finally, 138 pediatric ischemic-type MMD patients, including 34 who underwent DB and 104 who underwent IB, were retrospectively included in this study (Figure 1).

### Baseline Characteristics Before and After Propensity Score Matching

Clinical characteristics before and after propensity score matching were shown in Table 1. Before matching, patients underwent DB were significantly older than those who underwent IB (11.21 ± 4.10 vs. 9.56 ± 3.46 years old, *P* = 0.023). More patients were found with PCA involvement in the DB group than the IB group (14.7 vs. 35.6%, *P* = 0.037). The propensity score matching yielded 34 matched pairs with equivalent demographic and angiographic characteristics and these pairs were used for further analysis. Detailed baseline characteristics of the matched couples were outlined in Table 1.

**Table 1:** Patient characteristics and group comparisons before and after propensity score matching.

**Characteristics**	**Before propensity score matching**			After propensity score matching		
	**DB**	**IB**	***P*-value**	**DB**	**IB**	***P-*value**
No. of patients	34	104		34	34	
Age	11.21 ± 4.10	9.56 ± 3.46	0.023^*^	11.21 ± 4.10	11.26 ± 3.61	0.950
Male/female ratio	18:16	53:51	0.998	18:16	16:18	0.808
mRS score on admission						
0	2 (5.9)	3 (2.9)		2 (5.9)	2 (5.9)	
1	26 (76.5)	71 (68.3)	0.395	26 (76.5)	28 (82.4)	0.789
2	6 (17.6)	25 (24.0)		6 (17.6)	4 (11.8)	
3	0 (0.0)	5 (4.8)		0 (0.0)	0 (0.0)	
Cerebral infarction	9 (26.5)	28 (26.9)	1.000	9 (26.5)	9 (26.5)	1.000
Suzuki stage						
I	1 (2.9)	3 (2.9)		1 (2.9)	1 (2.9)	
II	2 (5.9)	4 (3.8)		2 (5.9)	3 (8.8)	
III	12 (35.3)	41 (39.4)	0.836	12 (35.3)	11 (32.4)	0.914
IV	14 (41.2)	33 (31.7)		14 (41.2)	12 (35.3)	
V	5 (14.7)	21 (20.2)		5 (14.7)	6 (17.6)	
VI	0 (0.0)	2 (1.9)		0 (0.0)	1 (2.9)	
Unilateral MMD	3 (8.8)	6 (5.7%)	0.821	3 (8.8)	1 (2.9)	0.606
PCA involvement	5 (14.7)	37 (35.6)	0.037^*^	5 (14.7)	7 (20.6)	0.750

* *P < 0.05*.

### Surgical Complications

The mean duration of surgery was shorter in IB than DB (138.2 ± 40.6 vs. 203.0 ± 50.9 min, *P* < 0.001, Table 2). Among 68 hemispheres which had undergone surgical revascularization, 9 (13.2%) had postoperative complications, with 6 (17.6%) occurred in the DB group and 3 (8.8%) occurred in the IB group [*P* = 0.476, power (1-β): 28.2%]. In the DB group, 1 (2.9%) hemispheres had new-developed infarction, 3 (8.8%) had transient neurological deficits and 2 (5.9%) had seizures. In IB group, the incidences of aforementioned complications were 1 (2.9%), 2 (5.9%), and 0 (0.0%), respectively (*P* > 0.999, *P* > 0.999, *P* = 0.493, respectively). No postoperative cerebral hemorrhage occurred in the whole series. At discharge, mRS scores were not significantly different between the two groups [*P* = 0.244, power(1-β): 95.8%].

**Table 2:** Surgical complications of matched couples.

	**DB**	**IB**	***P*-value**
Surgical duration, min	203.0 ± 50.9	138.2 ± 40.6	<0.001
Complications	6 (17.6%)	3 (8.8%)	0.476^a1^
Cerebral infarction	1 (2.9%)	1 (2.9%)	>0.999
Transient neurological deficits	3 (8.8%)	2 (5.9%)	>0.999
Seizures	2 (5.9%)	0 (0.0%)	0.493
Cerebral hemorrhage	0 (0.0%)	0 (0.0%)	NA
mRS at discharge			
0	5 (14.7%)	12 (35.3%)	0.244^a2^
1	29 (85.3%)	19 (55.9%)	
2	0 (0.0%)	3 (8.8%)	

*Statistical power (1-β): a1: 28.2%, a2: 95.8%*.

### Long-Term Outcome

**Figure 2:**
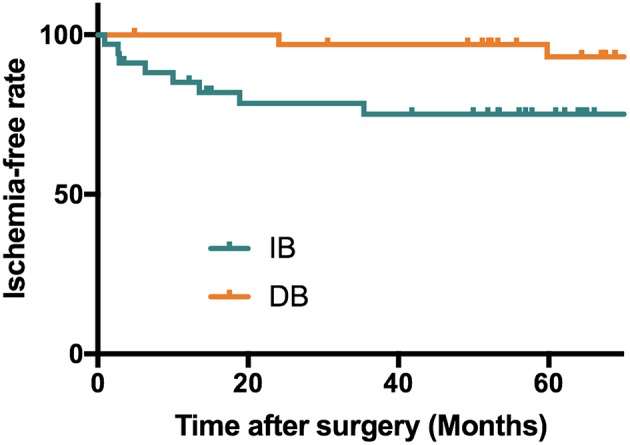
Kaplan-Meier analysis showing a longer stroke-free survival in patients underwent DB than patients who underwent IB (*P* = 0.025). Mean stroke free interval (months): DB: 70.2 ± 22.2 months; IB: 47.9 ± 31.2 months; Median stroke free interval: DB: 71.5 months; IB: 54.7 months.

**Figure 3:**
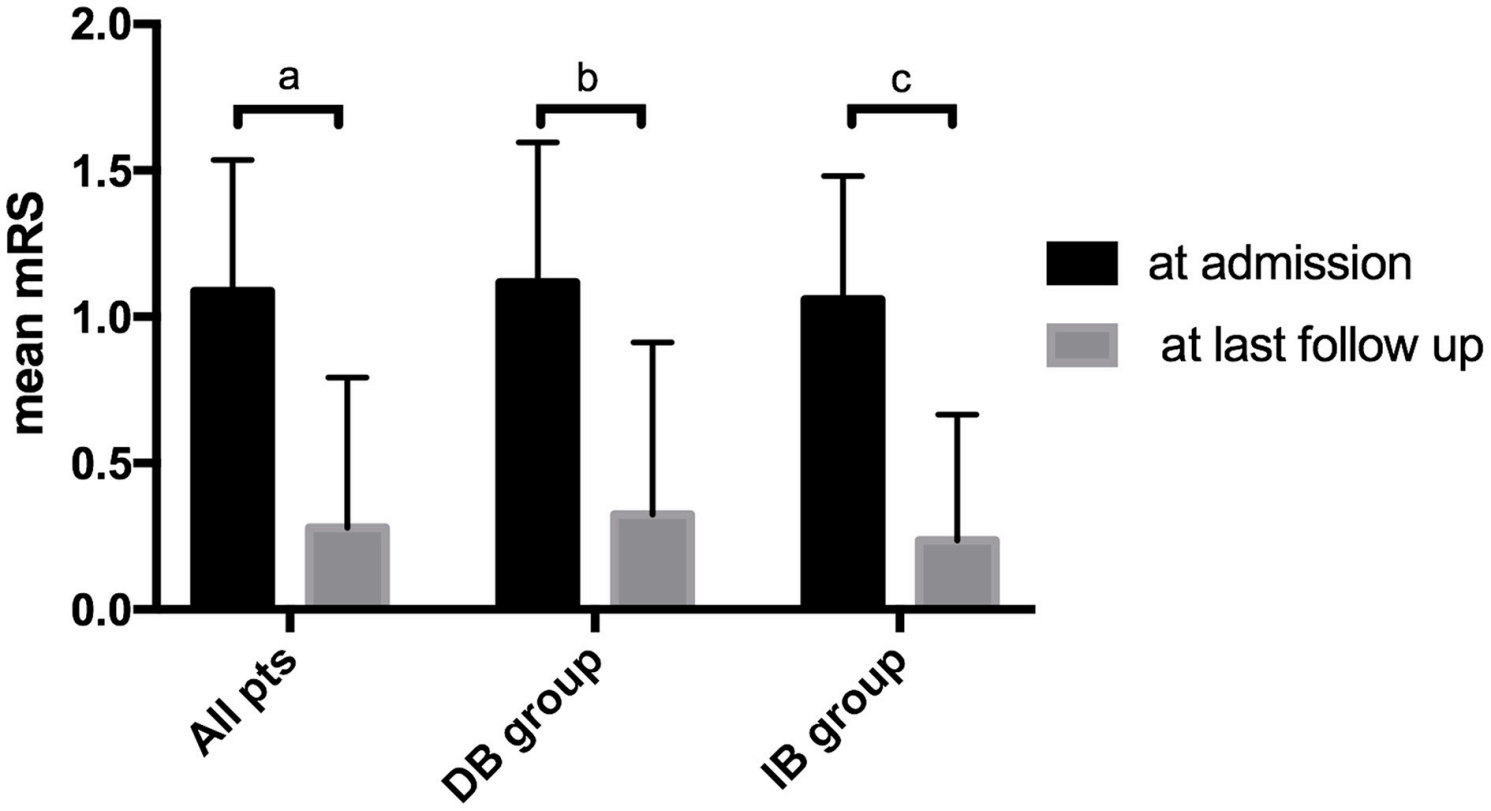
Comparison of patients' mRS score at time of admission and at last follow-up. a. Comparison of mRS in all patients at time of admission and at last follow-up (1.09 ± 0.45 vs. 0.28 ± 0.51, *P* < 0.001). b. Comparison of mRS in the DB group at time of admission and at last follow-up (1.12 ± 0.48 vs. 0.32 ± 0.59, *P* < 0.001). c. Comparison of mRS in the IB group at time of admission and at last follow-up (1.06 ± 0.42 vs. 0.24 ± 0.43, *P* < 0.001).

After propensity score matching, the DB group were followed for a mean of 71.9 ± 22.2 months, and the IB group were followed for a mean of 60.2 ± 24.3 months (*P* = 0.041). During follow-up, 10 (14.7%) patients had recurrent ischemic symptoms, including two cerebral infarctions and eight TIAs. Two (5.9%) ischemic events occurred in the DB group (TIA:1; cerebral infarction:1) and 8 (23.5%) occurred in the IB group (TIA:7; cerebral infarction:1). Kaplan-Meier analysis showed that patients underwent DB had a significantly longer stroke-free time than those underwent IB (*P* = 0.025, Figure 2). At the time of the last follow-up, good neurological status (mRS ≤ 1) was achieved in 32 (94.1%) of the DB group and 34 (100.0%) of the IB group (Table 3). Due to the significant difference of follow-up timing between the groups, mRS at last follow-up were not compared between DB and IB groups. MRS scores evaluated at the last follow-up were significantly lower than those at the time of admission in the entire group (1.09 ± 0.45 vs. 0.28 ± 0.51, *P* < 0.001), in the DB group (1.12 ± 0.48 vs. 0.32 ± 0.59, *P* < 0.001), and in the IB group (1.06 ± 0.42 vs. 0.24 ± 0.43, *P* < 0.001, Figure 3).

**Table 3:** Long-term follow-up of matched couples.

	**DB**	**IB**
Follow up, months^*^	71.9 ± 22.2	60.2 ± 24.3
mRS at last follow-up		
0	25 (73.5%)	26 (76.5%)
1	7 (20.6%)	8 (23.5%)
2	2 (5.9%)	0 (0.0%)

* *Follow-up duration between the DB and the IB groups were significantly different (P < 0.05)*.

## Discussion

Both DB and IB have been applied in the revascularization for pediatric MMD patients and received positive effects (5, 7, 9). It has been proposed that DB for children is technically more challenged due to the smaller size of vessels and presumably riskier during perioperative period (11, 13, 14), while IB, being a much simpler technique, provides sufficient revascularization for pediatric MMD with fewer postoperative risks (19). However, a consensus has not been reached regarding which revascularization surgery is the most beneficial for pediatric patients in the long-term. In this study, we retrospectively reviewed pediatric patients who underwent DB and IB at our center, using propensity score matching to reduce the heterogeneity between groups, and comparing surgical complications and long-term outcomes between pediatric MMD patients received different surgical modalities.

After propensity score matching, 68 pediatric ischemic-type MMD patients (34 matched pairs) were included in this study, with one DB and one IB procedure in each group. Our results showed although the procedure of DB requires a longer time than IB (203.0 ± 50.9 vs. 138.2 ± 40.6 min, *P* < 0.001), the overall incidences of postoperative complications were not significantly different between the two groups [17.6 vs. 8.8%, *P* = 0.476, power (1-β): 28.2%]. Previous studies have suggested that for adult MMD patients, DB, though being technically challenged, did not bring more postoperative risks (9, 18), however, this viewpoint had not been verified in pediatric patients. Hayashi et al reported a rather high incidence of postoperative neurological deterioration (15 in 35 operations, including two permanent neurological deficits) in pediatric MMD following DB, raising the concern of the potential higher risk direct bypass might bring to pediatric patients (20). However, in more recent studies, such a high incidence of postoperative ischemic events has rarely been reported, probably due to the refinement of techniques and management strategies. Also, Rashad et al. pointed that proper blood pressure control might reduce the risk of hemodynamic disorders, therefore lower the incidence of postoperative complications (7, 14). Our findings showed no significant difference between the complication rates between DB and IB for pediatric MMD patients (17.6 vs. 8.8%, *P* = 0.476). However, the low sample size in the current study could impair the statistical power, which in this case, is 28.2%, therefore, there might be a chance that though DB is riskier than IB, the current study wouldn't be able to detect it. It is worth noticing that in our study, DB had more postoperative seizures and transient neurological deficits than the IB group (Table 1) despite no significant difference was noted, although it definitely needs future studies with larger sample size to further validate the risk of complication after DB and IB procedures.

In this series, recurrent stroke events during follow-up were observed in 10 of 68 patients, with a pooled incidence of 14.7%, including 2 (2.9%) cerebral infarctions and 8 (11.7%) TIAs. The incidence of recurrent ischemic strokes was higher in the IB group (23.5%) than DB (5.9%) group, and Kaplan-Meier analysis showed a significant longer stroke-free time in the patients who underwent DB than those underwent IB (*P* = 0.025, Figure 2). The long-term effect of DB and IB had been investigated in MMD patients by a variety of studies, however, a comparison of the advantage and shortage between these two procedures for pediatric MMD patients had rarely been demonstrated. Our findings were in accordance with previous studies of adult MMD patients showing DB was superior to IB in preventing recurrent ischemic strokes (21–23), and suggested the same effect of DB than IB in pediatric MMD patients as well.

Having said that, our results also showed both DB and IB were effective in improving neurological status of pediatric MMD patients. MRS scores were significantly improved in the long-term follow-up after revascularization surgery, regardless of specific surgical modality (Figure 3). At last follow up, good neurological status (mRS ≤ 1) were achieved in the majority of both DB (94.1%) and IB (100.0%) group (Table 3), even with the higher incidence of recurrent ischemic events in the IB group. Unfortunately, due to the uneven follow-up duration, mRS scores were not compared between the groups for fear of incompatibility. Previous literature demonstrated that long-term functional outcomes were not significantly different between surgical modalities, despite the better revascularization provided by DB and combined revascularization (5, 24). This might be related to the relatively higher incidence of TIAs, which did not lead to significant neurological deterioration of neurological status, than cerebral infarctions during follow-up. Also, as demonstrated by a previous study by Uchino et al., in the combined bypass surgery for MMD, DB might undergo reciprocal regression during the chronic development of spontaneous collaterals development. As they suggested, IB might be the dominant blood supply in the long term (25), which probably explained for the similar effect of DB and IB on the improvement of neurological functions.

In this study, we used propensity score matching to reduce the heterogeneity between patients who underwent different surgical revascularizations. The variables that we unified were mainly conditions and characteristics of patients when they had surgery, however, one factor that cannot be matched, but should not be ignored was the surgeon's experience, which might significantly affect patients' outcomes, especially in DB procedure. The DB bypasses we included in the current study were performed by one experienced surgeon, therefore reducing the potential bias that it may cause on the long-term outcome.

This study had some limitations. First, the study was retrospectively designed. Although propensity score matching was used to reduce the heterogeneity that resulted from non-randomization, potential selection bias and confounders could not be totally excluded. Second, the sample size of this study was rather small, especially after propensity score matching, therefore the statistical power might be compromised. Third, the follow-up was conducted mainly by telephone interviews, which is the most available method but could be less reliable. Pediatric patients might forget episodes of TIAs, and sides and extremities involved at the time of ischemic episodes. The lack of long-term follow-up radiological imaging (MRI or CT) made it unable to detest silent strokes. However, with previously limited knowledge of risks and benefits, we were not able to proceed a randomized trial directly, therefore, a study with matched couples might currently be the most suitable method for this investigation. Future studies are still needed to confirm the effect of DB and IB for pediatric MMD patients.

## Conclusion

Both direct bypass and indirect bypass are effective in improving the neurological status of pediatric ischemic-type MMD patients, and direct bypass might be more superior in preventing recurrent ischemic strokes.

## Author Contributions

YahZ and XD: conception and design. YahZ, SY, JLu, and JLi: acquisition of data. YahZ: analysis and interpretation of data and drafting the article. YanZ, DZ, RW, HW, and YuaZ: technical supports and surgery. YuaZ: study supervision and approved the final version of the manuscript on behalf of all authors. All authors: critically revising the article and reviewed submitted version of manuscript.

### Conflict of Interest Statement

The authors declare that the research was conducted in the absence of any commercial or financial relationships that could be construed as a potential conflict of interest.
